# Meteorological and social conditions contribute to infectious diarrhea in China

**DOI:** 10.1038/s41598-021-00932-0

**Published:** 2021-12-03

**Authors:** Xiang Yang, Weifeng Xiong, Tianyao Huang, Juan He

**Affiliations:** 1 Beijing University of Chinese Medicine, No. 11, Bei San Huan Dong Lu, Chaoyang District, Beijing, 100029 China; 2 Tsinghua University, Haidian District, Beijing, 100084 China

**Keywords:** Disease prevention, Risk factors

## Abstract

Infectious diarrhea in China showed a significant pattern. Many researchers have tried to reveal the drivers, yet usually only meteorological factors were taken into consideration. Furthermore, the diarrheal data they analyzed were incomplete and the algorithms they exploited were inefficient of adapting realistic relationships. Here, we investigate the impacts of meteorological and social factors on the number of infectious diarrhea cases in China. A machine learning algorithm called the Random Forest is utilized. Our results demonstrate that nearly half of infectious diarrhea occurred among children under 5 years old. Generally speaking, increasing temperature or relative humidity leads to increased cases of infectious diarrhea in China. Nevertheless, people from different age groups or different regions own different sensitivities to meteorological factors. The weight of feces that are harmfully treated could be a possible reason for infectious diarrhea of the elderly as well as children under 5 years old. These findings indicate that infectious diarrhea prevention for children under 5 years old remains a primary task in China. Personalized prevention countermeasures ought to be provided to different age groups and different regions. It is essential to bring the weight of feces that are harmfully treated to the forefront when considering infectious diarrhea prevention.

## Introduction

Infectious diarrhea is a preventable disease yet remains a major public health problem around the world^1^. According to World Health Organization, diarrheal disease accounts for approximately 1.8 million deaths each year and is the second leading cause of death in children under five years old, which is responsible for killing around 0.525 million children every year. Developing countries suffer much more badly from infectious diarrhea^2,3^. China is a middle-income country with vast territory and varied climates. The Chinese government provides heavily subsidized healthcare as well as wide public health education, yet still endures significant diarrhea burden. China ranks among the top 15 countries on the number of diarrheal morbidity^4^. In China, the infectious diarrhea cases account for nearly 20% of those of 45 notifiable infectious diseases during the last 10 years^5^. Despite the high burden, our ability to predict and prepare for diarrheal case surges remains limited^6,7^.

Researches have found out that the incidence of infectious diarrhea has a very close relationship with seasonal change for decades^8–12^, in particular with meteorological conditions^13,14^. In general, various studies done in different parts of the world suggest that increasing temperature or relative humidity leads to increasing infectious diarrhea cases^15–24^, only with a few exceptions^25,26^. Extreme weather events such as floods or El Nino warm events greatly trigger infectious diarrhea in various localities^14,21,27–31^. For instance, higher maximum temperature and more sunshine in the pre-monsoon period are found to have a tendency to enhance the first peak of the diarrhea occurrence in tropical area^32^. Climate was considered to dominate high- and low-bacterial enteric diseases periods in Vietnam^33^. Higher ambient temperature decreases and higher rainfall increases diarrheal risk in rural sites of south India^34^. Etiological and meteorological factors had age-specific effects on the prevalence of infectious diarrhea in Jiangsu province of China^35^. La Nina conditions lagged 0–5 months are associated with higher than average incidence of under-age-5 diarrhea in the early rainy season in Botswana^36^.

In addition to the meteorological conditions, other studies illustrate that some social conditions also contribute to incidence of infectious diarrhea^24,37–40^. Those social factors can be classified into 4 categories. The first one is the resource of public health, like medical and technical personnel^41^, access of hygienic conditions^42–44^ and nutrition status^45–49^. The second one is the safety of water, in particular, the quality and quantity of clean water available for cooking, drinking and washing^42,50–53^. The third one is the fecal contamination, like the septic system densities^51^, access to private sanitation^54^. The fourth one is the local forest coverage^24,55^. For instance, it has been found that medical and technical personnel per thousand persons were significantly negatively related to the risk of bacterial dysentery in Sichuan province of China^41^. Use of river water for cooking and cleaning was highly associated with risk of typhoid fever in Blantyre, Malawi^53^. Septic system densities were associated with infectious diarrhea in central Wisconsin^51^. Local forest coverage may mitigate the effects of extreme heat on cholera outbreaks in Bangladesh^24^.

While the impacts of potential factors on diarrheal incidence have been well documented, existing researches generally suffer from three drawbacks. First, those researches mainly focused on only one kind of infectious diarrhea^56^. Second, social factors that might contribute to statistical incidence of infectious diarrhea have not been put on enough attention^24,38,39^. Third, with regard to the fact that the inter-relations among the predominant factors of infectious diarrhea are quite complicated in the real world, traditional statistics methods could be unsuitable for discovering these complex relationships^22,57–60^.

In this study, we explore the link between monthly/yearly variability and all sorts of infectious diarrhea during 2005-2017 in China. The potential factors that we take into consideration are meteorological and social factors. The method for data analysis is a well established algorithm in the field of machine learning. The infectious diarrhea cases that have been reported to Chinese Center for Disease Control and Prevention (China CDC), are grouped into three classes: Class A (cholera); Class B (bacillary dysentery, typhoid and paratyphoid); and Class C (other infectious diarrhea). We apply some meteorological factors that are the most commonly used factors^11,12^, including precipitation, wind speed, temperature, vapor pressure, and relative humidity.

Based on existing researches, we apply 4 categories of social factors to explore the linkage between social factors and infectious diarrhea. The factors we choose are the representative ones for each kind. In specific, we choose the quantity of centers for disease control and prevention (CDCP), the quantity of health supervision institute (HSI) and the quantity of health technicians (HT) to represent the resource of public health. As water pollution is usually the main threat to the safety of water^61^, the three major measuring elements of wastewater discharge are utilized to represent the safety of water. Those three elements are the amounts of wastewater discharge (WD), chemical oxygen demand emissions (CODE) and nitrogen output (NO). The weight of feces that are harmfully treated (FHT) is selected to represent the severity of fecal contamination. And the forest coverage rate (FCR) is used to represent the local forest coverage. The data we use in this study are all publicly available.

## Results

### Epidemiology of infectious diarrhea

A total of 14,396,560 cases of infectious diarrhea were reported during 2005–2017 in mainland of China. Cases aged under 5 years accounted for 46.78% (6,733,533/14,396,560) of all cases.

**Figure 1:**
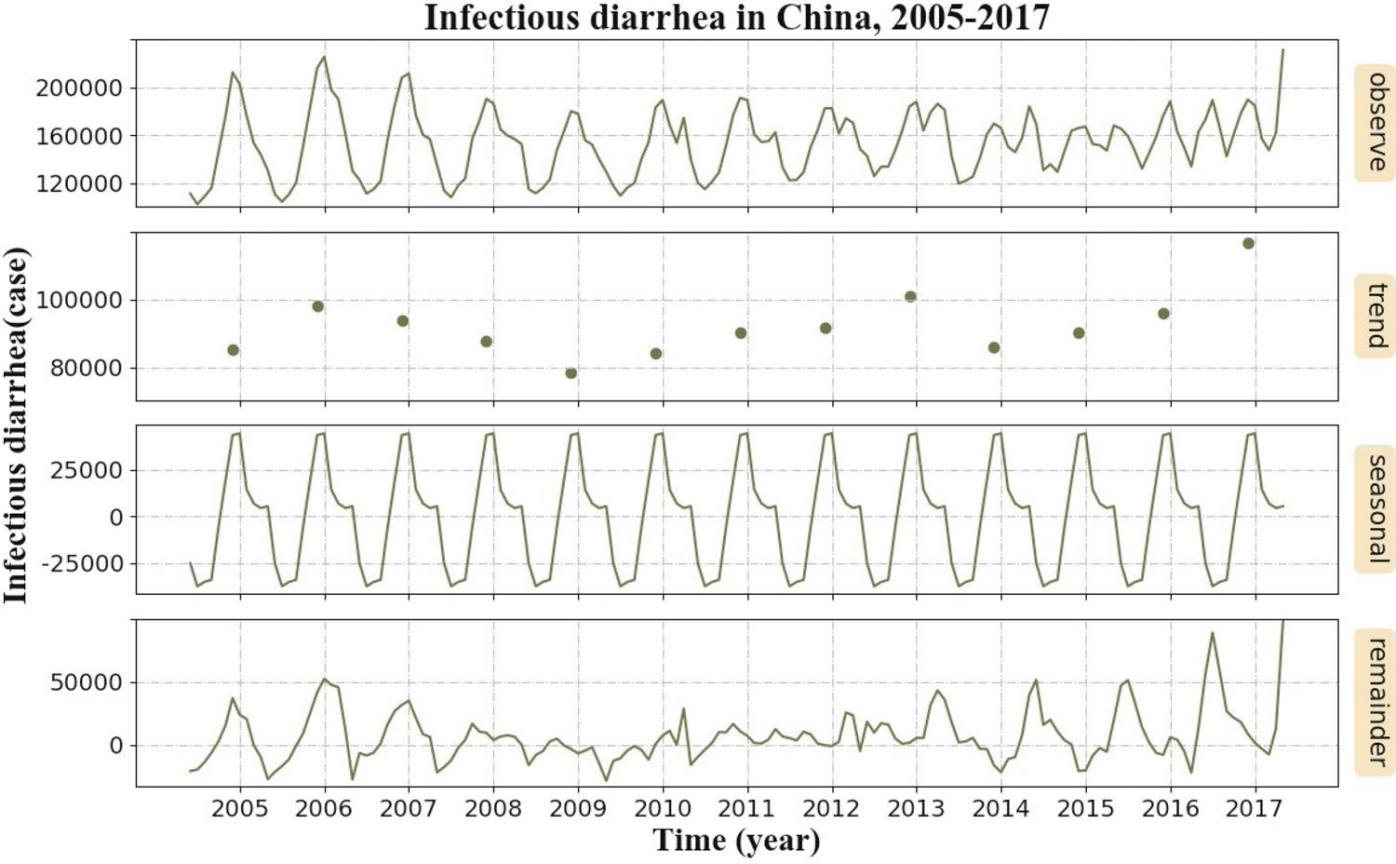
Monthly observed cases of infectious diarrhea in mainland of China, 2005–2017. Note: From top to bottom, the lines represent actually observed cases, trend components, seasonal components, and random components, respectively.

The overall situation of infectious diarrhea in mainland of China is shown in Fig. 1. The first pane in the figure is the monthly observed number of infected cases during 2005–2017. The second pane is the average number of infected diarrhea cases of the whole year, which represents the infectious diarrhea trend with time. The third pane considers the average number of infected cases of the specific month during the observed 13 years, which is applied to represent the seasonal pattern of infectious diarrhea. These monthly averages are then normalized by subtracting the mean of these averages. The fourth pane is the remained data, which are obtained by the observed number minus both the yearly and monthly averaged numbers. The corresponding month of each year labeled in the horizontal ordinate is July. The cases indicated a long-term increasing trend, which is shown in the second pane. In the third pane, a peak from May to August and a nadir from November to February are observed annually.

**Figure 2:**
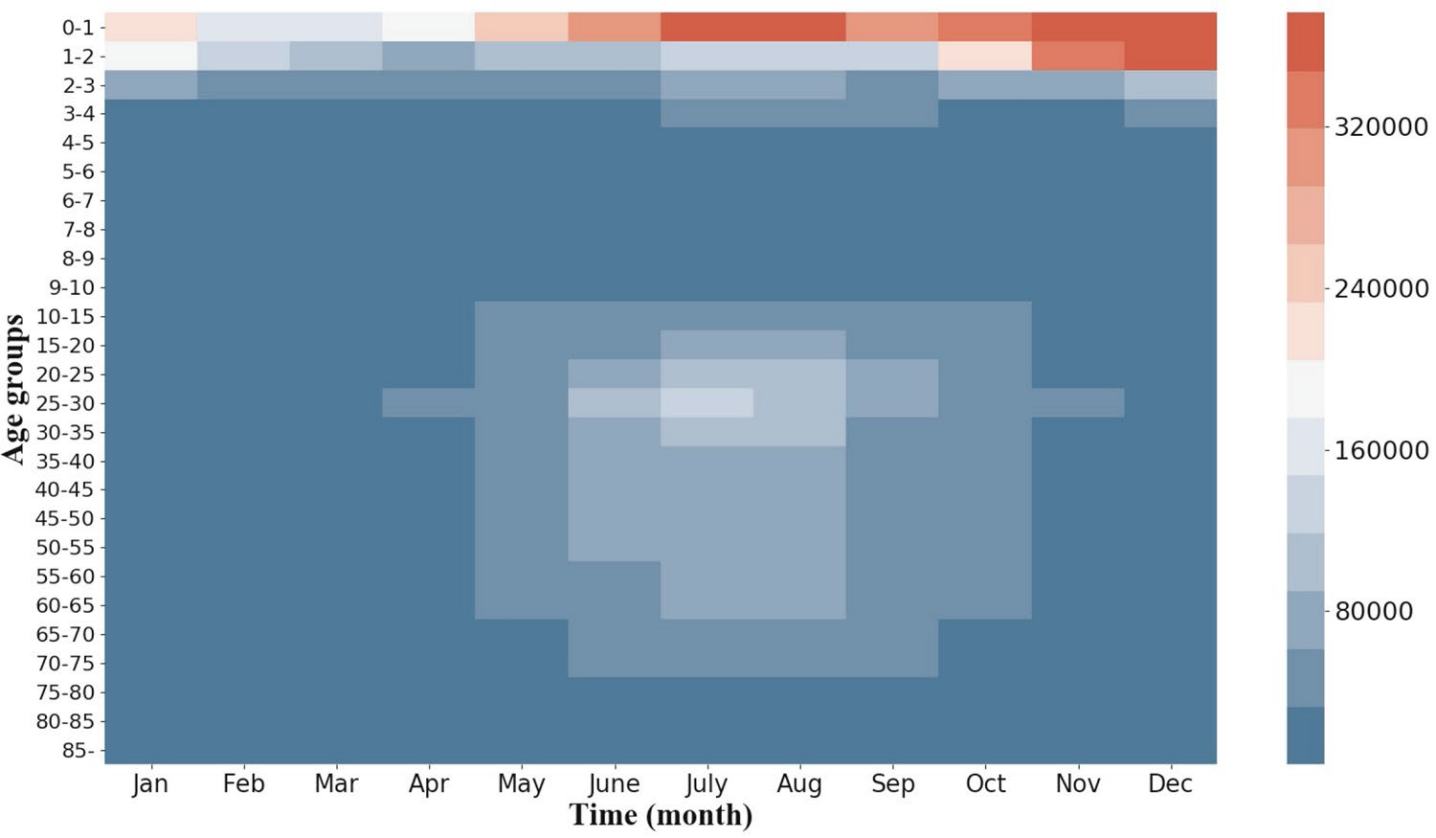
Seasonal and age-specific numbers of infectious diarrhea cases in mainland of China, 2005–2017. (See Supplementary Table 1 in the Supplementary material to obtain the detailed data). The heat map is created by package seaborn in Python^62^.

The seasonal, age-specific numbers of infectious diarrhea cases are demonstrated in Fig. 2. From this figure, we observe that the population clustered into three age groups: under 5, 5–10, and over 10 years age. The incidence of infectious diarrhea in the group with age under 5 years old increased mainly in the early autumn and winter, while the incidence in the group with ages over 10 years old increased mainly in summer. In comparison, the incidence of infectious diarrhea in the 5–10 age group stayed quite stable and was less sensitive to season change.

**Figure 3:**
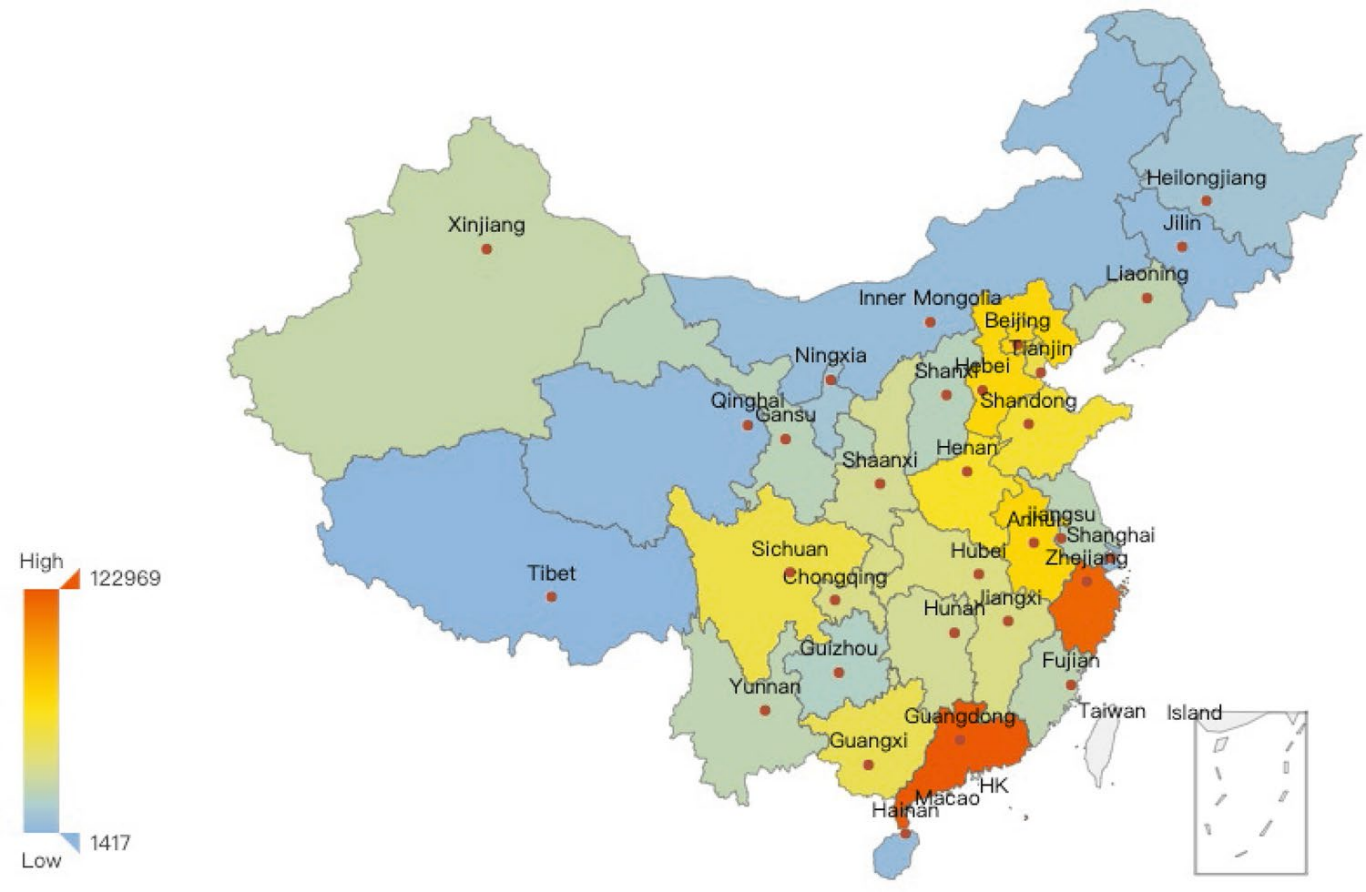
The yearly averaged numbers of infectious diarrhea cases in each province of mainland of China during 2005–2017. The map is generated by an open source package called pyechart in Python^63^.

**Figure 4:**
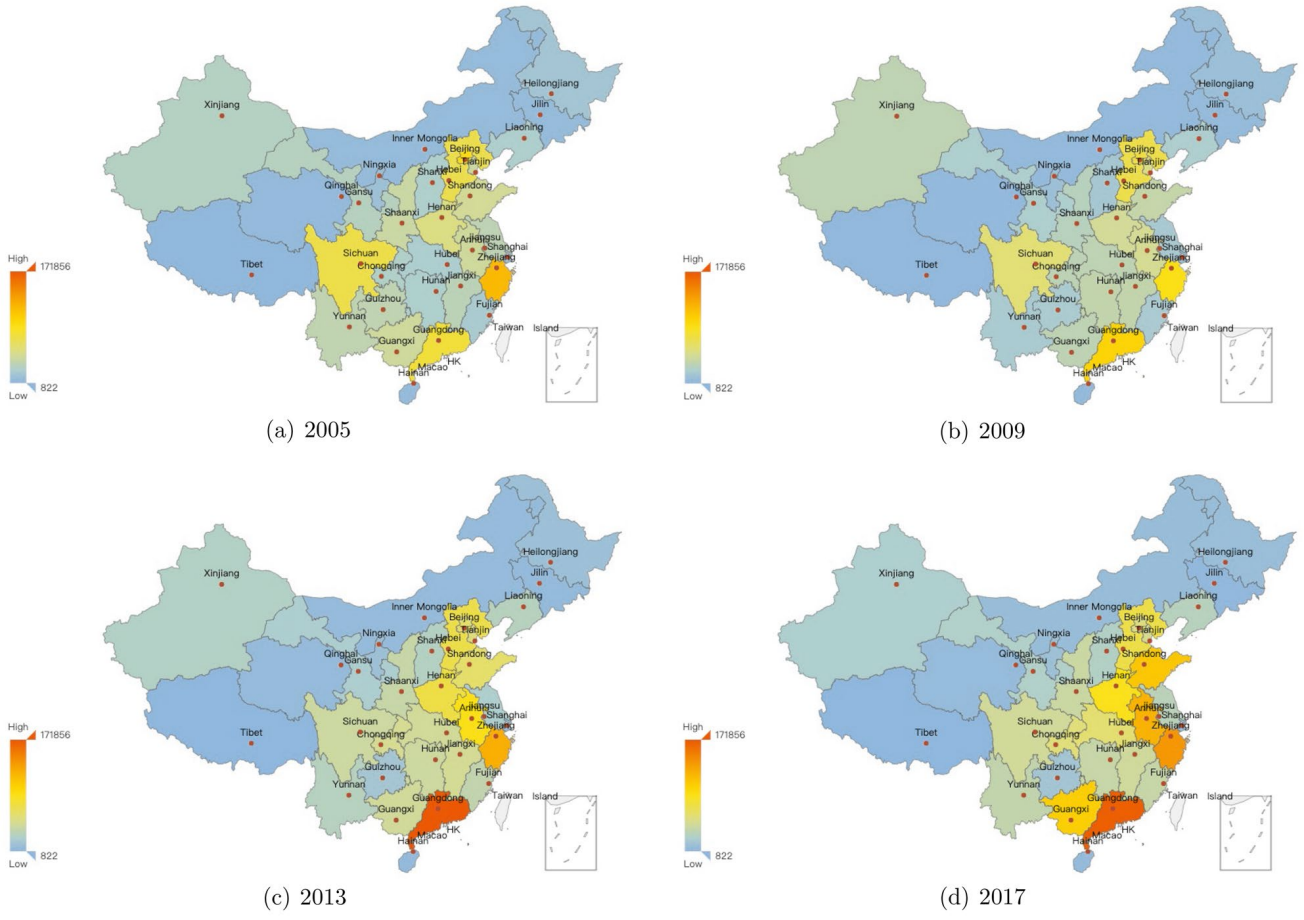
Provincial numbers of infectious diarrhea cases in mainland of China, in four different years during 2005–2017. The map is generated by an open source package called pyechart in Python^63^.

**Figure 5:**
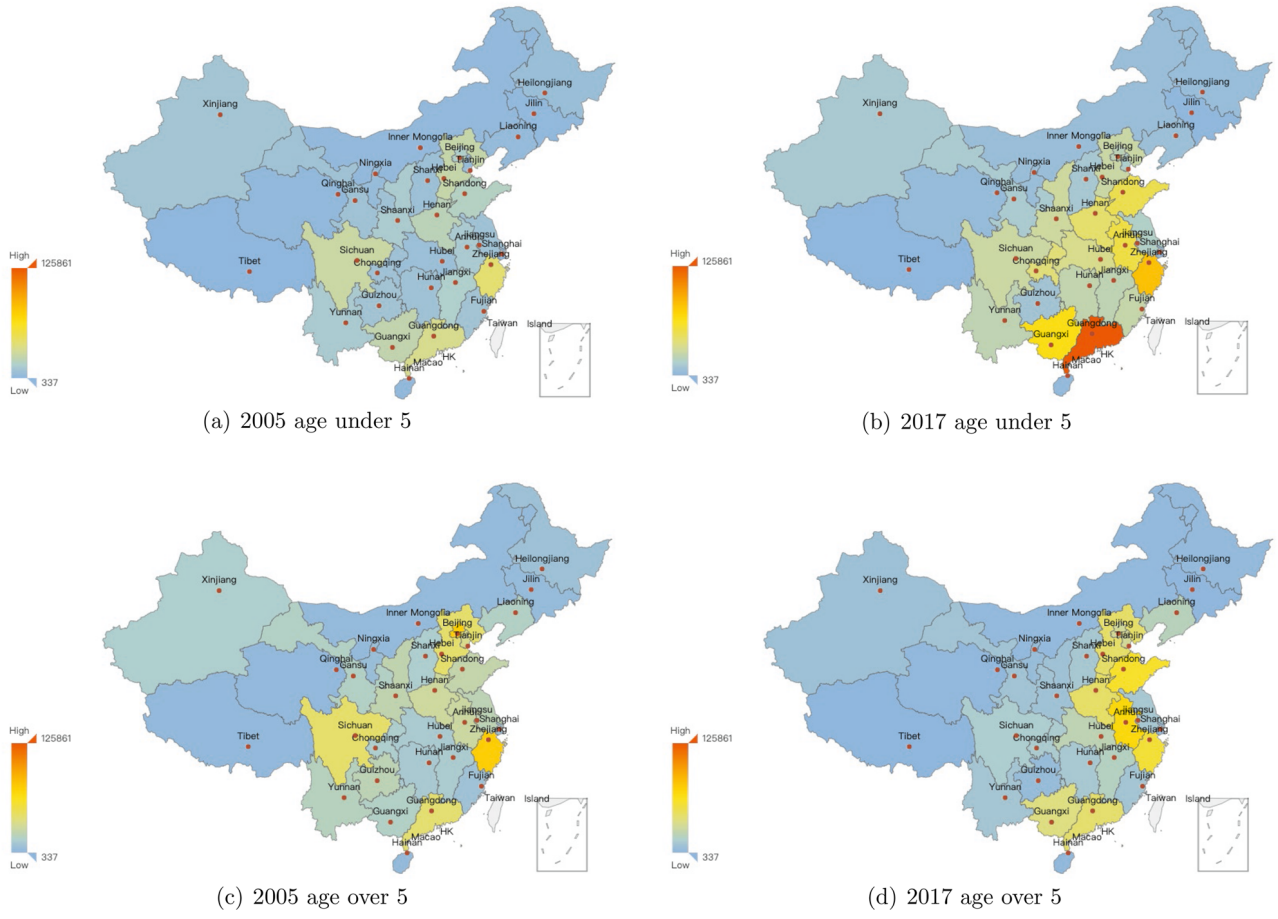
Provincial numbers of infectious diarrhea cases with regard to different age groups in mainland of China, in 2005 and 2017. The age groups are divided into two: under and over 5 years. The map is generated by an open source package called pyechart in Python^63^.

The provincial situations about yearly infectious diarrhea cases are demonstrated in Figs. 3, 4, 5. The yearly averaged number of infectious diarrhea cases in mainland of China during 2005–2017 (shown in Fig. 3) illustrates significant differences between provinces. The top 4 provinces with the most infected cases are Guangdong, Zhejiang, Anhui and Shandong provinces. Figure 4 provides provincial numbers of cases in four concrete years (2005, 2009, 2013 and 2017), indicating that the number of infectious diarrhea cases increased in most of the provinces during 2005–2017. The primary cases lied in southeast coastal provinces when considering the increase of all age groups. Figure 5 focuses on two age groups: under and older than 5 years old, and demonstrate the provincial number of infectious cases in 2005 and 2017. The increased cases of under 5 years old are majorly distributed in Guangdong and Zhejiang provinces. For the age group over 5 years old, the increased cases are mainly distributed in Anhui province. Only among the over-5 age group we observe the reduced number of infectious diarrhea cases, which happened in Beijing and Zhejiang province.

### The influence of social factors with regard to different age groups

**Figure 6:**
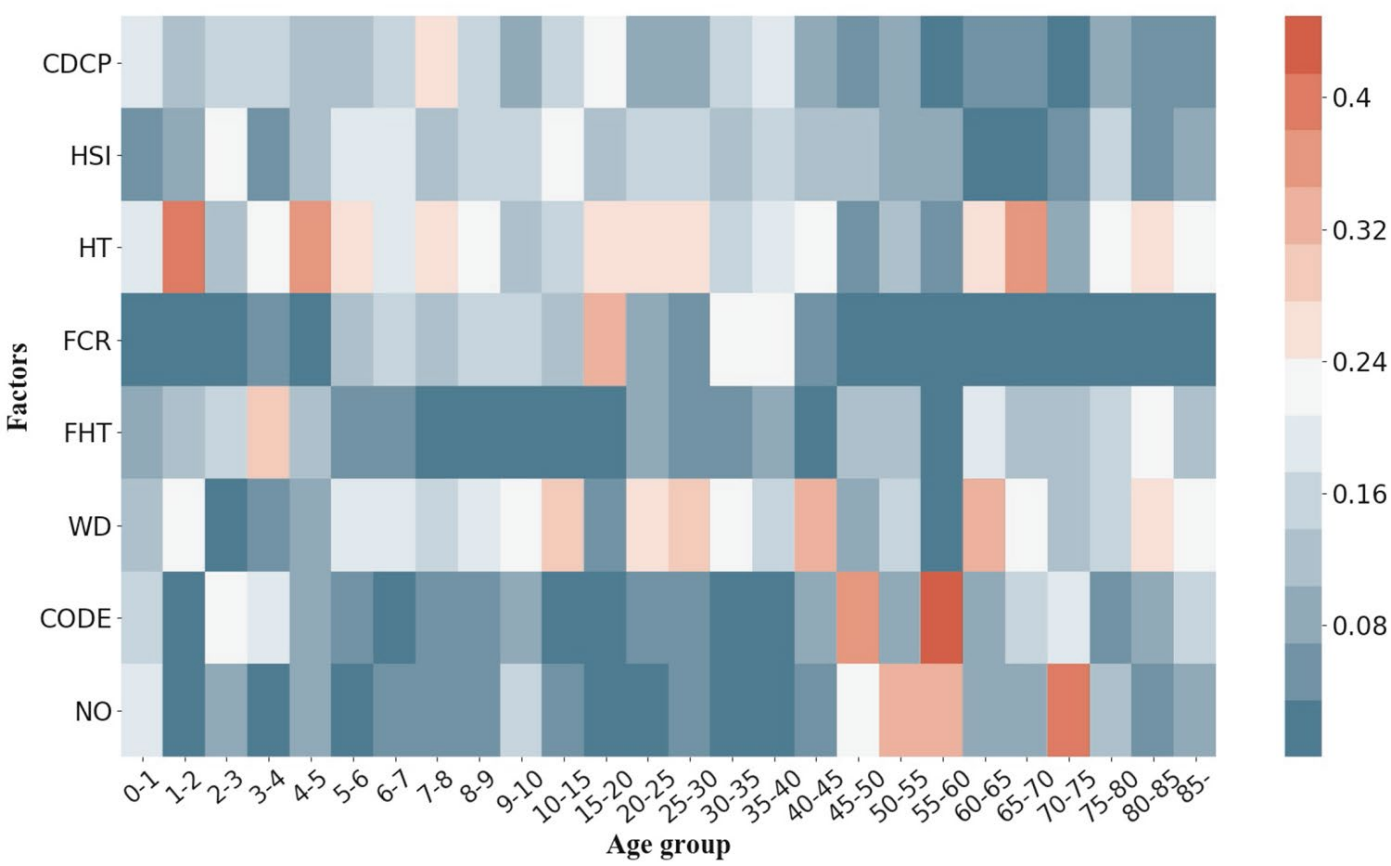
Heat maps of influence of social features on the number of infectious diarrhea cases in different age groups in mainland of China, from 2005 to 2017. (See Supplementary Table 2 in the Supplementary material to obtain the detailed data). The heat map is created by package seaborn in Python^62^.

The social factors we take into consideration are: the quantity of centers for disease control and prevention (CDCP), the quantity of health supervision institute (HSI), the quantity of health technicians (HT), the amount of wastewater discharge (WD), chemical oxygen demand emissions (CODE), nitrogen output (NO), the weight of feces that are harmfully treated (FHT), and the forest coverage rate (FCR). The yearly data of social factors as well as the number of infectious cases in different age groups are illustrated later in Table 1. The influence of social factors on the number of infectious diarrhea cases in different age groups is shown in Fig. 6 and we leave the detailed data in Supplementary Table 2 of the Supplementary material.

**Table 1:** Yearly data of number of infectious diarrhea cases in two age groups, as well as the social factors.

Year	# of cases		CDCP^a^	HSI^b^	HT^c^	POFC^d^	FHT^e^	TWD^f^	CODE^g^	NO^h^
	$\hbox {age}<5$	$\hbox {age}>=5$								
2005	366,976	656,625	3585	1702	456.41	18.2	1090.8	5,245,089	1414.2	149.78
2006	417,873	759,072	3548	2097	472.84	18.2	1275.3	5,144,802	1414.2	149.78
2007	449,620	676,076	3585	2553	491.32	18.2	1598	5568494.16	1381.8	132.34
2008	473,458	580,909	3534	2675	517.45	20.4	1426.8	5,716,801	1320.7	126.97
2009	415,755	523,964	3536	2809	553.51	21.6	1294.9	5890877.25	1277.5	122.61
2010	479,839	528,847	3513	2992	587.62	21.6	1259.7	6,172,562	1238.1	120.29
2011	507,217	576,101	3484	3022	620.29	21.6	1310.1	6591922.44	2499.86	260.44
2012	521,157	580,842	3490	3088	667.55	21.6	1010.4	6847612.14	2424	253.59
2013	640,027	572,002	3516	2967	721.06	21.6	1004.6	6954432.7	2352.7	245.66
2014	517,576	514,027	3490	2975	758.98	21.6	860	7161750.53	2294.6	238.53
2015	555,034	530,361	3478	2986	800.75	21.6	763.1	7353226.83	2223.5	229.91
2016	587,744	562,750	3481	2986	845.44	21.6	652.1	7110953.88	1046.53	141.78
2017	801,257	601,451	3456	2992	898.82	21.6	540	6996609.97	1021.97	139.51

^a^The quantity of centers for disease control and prevention.

^b^The quantity of health supervision institute.

^c^The quantity of health technicians (10,000).

^d^The forest coverage rate (%).

^e^The weight of fecal harmful treatment (10,000 tons).

^f^The amounts of wastewater discharge (10,000 tons).

^g^Chemical oxygen demand emissions (10,000 tons).

^h^Nitrogen output (10000 tons).

Generally speaking, social factors have different influence on infectious diarrhea cases of different age groups in mainland of China. Some age groups have principal influential factors while other age groups do not. For adolescents with age between 10 and 25, HT is the dominant factor. For middle aged people with years between 45 and 60, NO seems to affect those group more. For elderly aged over 75, WD matters the most.

From the perspective of social factor, CDCP, HSI, HT and WD affect the number of infectious diarrhea cases in almost the whole age groups. FCR has more significant impact on ages between 5 and 40 than other age groups. FHT primarily strikes the old and young. CODE has a main contribution in age groups of 45–50 and 55–60. NO has the most clear influence to people between 70 and 75 years old.

### Predicting future numbers of infectious diarrhea cases by meteorological and social factors

In this article, we adopt meteorological factors and social factors to predict future number of infectious diarrhea cases and get reasonable results. The monthly data of infectious diarrhea cases in mainland of China during 2005–2015, which were divided by age groups and provinces, are adopted as training data. The rest data of 2016-2017 are adopted as prediction test data.

When predicting future infectious diarrhea cases, we use an existing package called scikit-learn in Python to implement Random Forest regression. For each province in mainland of China, we divided the diarrheal patients into 26 age groups (0–1, 1–2, 2–3, 3–4, 4–5, 5–6, 6–7, 7–8, 8–9, 9–10, 10–15, 15–20, 20–25, 25–30, 30–35, 35–40, 40–45, 45–50, 50–55, 55–60, 60–65, 65–70, 70–75, 75–80, 80–85, 85-). For each age-province combination, we apply the cross_val_score function in scikit-learn to tune the parameters by 5-fold cross validation. The parameters being tuned are the number of trees in the forest (tuning scale: 50–300, tuning step:10) and the maximum depth (tuning scale: 5–30, tuning step:2), while other parameters are set to the default values (see Supplementary Table 3 in Supplementary material to obtain the tuned parameters for each age-province combination and Supplementary Table 4 to obtain the exhaustive list of other parameters). After the parameter tuning, we run 10 times of train-test implementation and obtain the mean error and an indication of the variance around that mean (see Supplementary Table 5 in Supplementary material to obtain the detailed data).

We apply a method called ${\mathrm {nMAE}}$ as predicting evaluation, which is a normalized metric, defined by^64^:

1 $$\begin{aligned} {\mathrm{nMAE}} = \frac{\mathrm{MAE}}{ \bar{x}}. \end{aligned}$$

Here, $\bar{x}$ is the average value of *x*, and ${\mathrm {MAE}}$ is the mean absolute error, which is defined by:

2 $$\begin{aligned} {\mathrm{MAE}} = \frac{1}{N} \times \sum _{i=1}^N \big | {\mathrm{predicted}}_i -{\mathrm{true}}_i \big |. \end{aligned}$$

**Figure 7:**
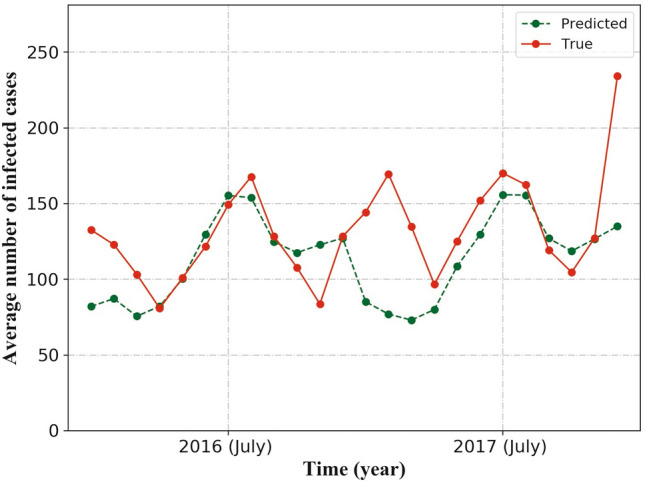
The comparison between average number of cases in the predicted data and the counterparts in the true data. The red line stands for true value while the green line stands for predicted value. (See Supplementary Table 6 in Supplementary material to obtain the detailed data).

**Figure 8:**
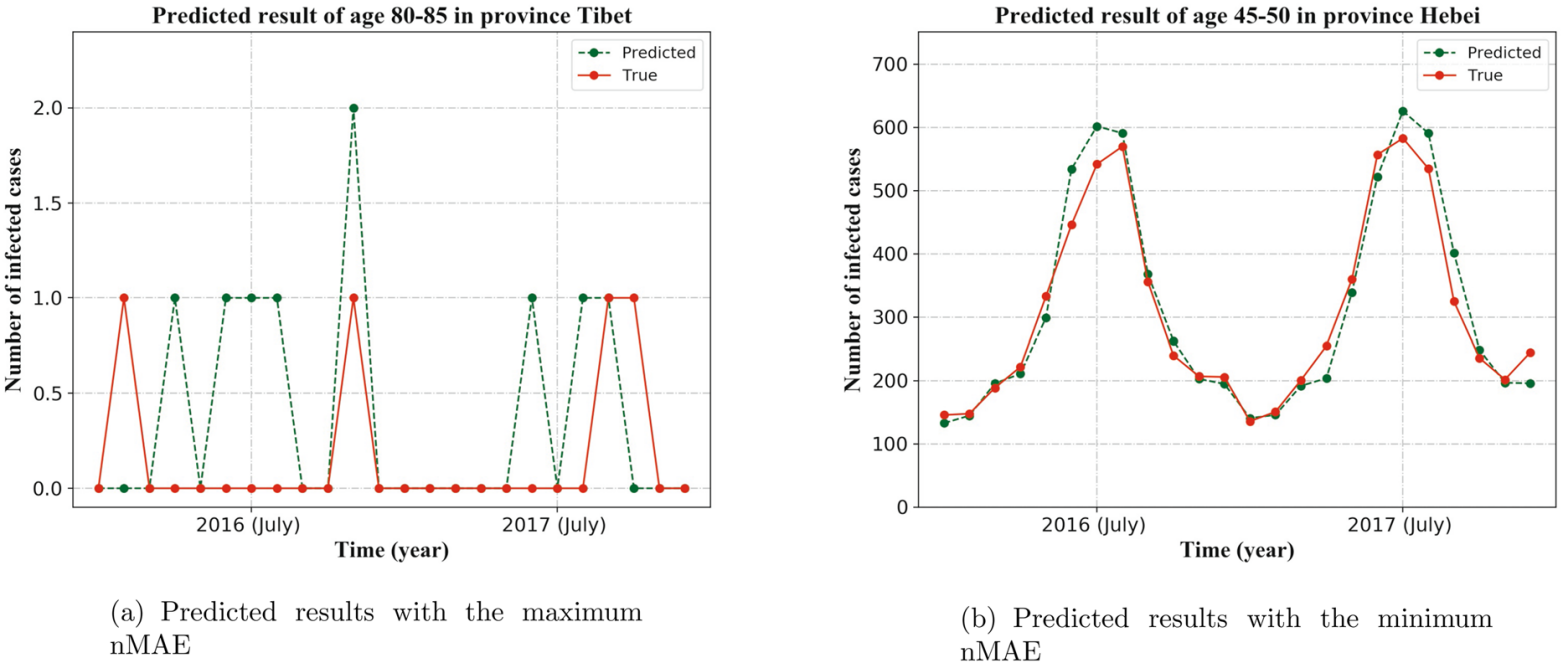
Demonstration of worst and best predictions. (See Supplementary Tables 7, 8 in Supplementary material to obtain the detailed data). The predicted result is measured by nMAE.

**Figure 9:**
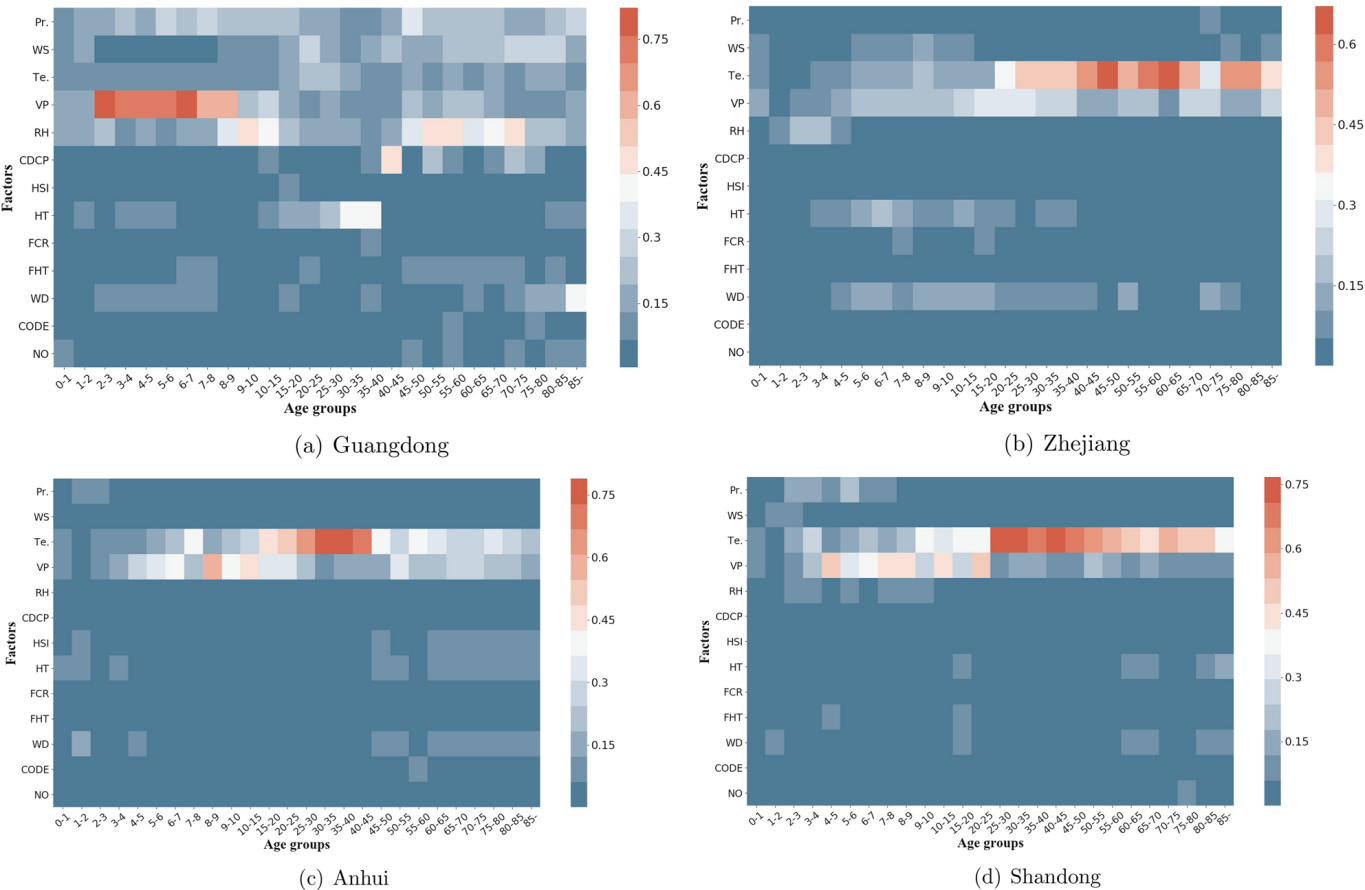
Heat maps of influence of potential factors on number of infected diarrhea cases of different age groups in the top four provinces with most infectious cases in mainland of China, from 2005 to 2017. (See Supplementary Tables 9, 10, 11, 12 in Supplementary material to obtain the detailed data). The heat map is created by package seaborn in Python^62^. Meteorological factors Pr.: precipitation, WT: wind speed, Te.: temperature, VP: vapor pressure, RH: relative humidity Social factors CDCP: the quantity of centers for disease control and prevention, HSI: the quantity of health supervision institute, HT: the quantity of health technicians, WD: the amounts of wastewater discharge, CODE:chemical oxygen demand emissions, NO: nitrogen output, FHT: the weight of feces that are harmfully treated, FCR: forest coverage rate.

Approximately, the average numbers of predicted infectious diarrhea cases coincide with those of the real situation in twelve months, which are April to October, December of 2016 and July to September, November of 2017 (shown in Fig. 7). In those twelve months, the difference between predicted data and real data are less than 10%. There are large gaps between predicted data and real data in seven months, which are January, February and November of 2016, and January to March, December of 2017. In those seven months, the difference between the two groups of data are more than 30% (see Supplementary Table 6 in Supplementary material to obtain the detailed data). The best predicting result (${\mathrm{nMAE}}=0.088$) and the worst predicting result (${\mathrm{nMAE=2.65}}$) are shown in Fig. 8). The detailed data about the worst and best predictions are given by Supplementary Tables 7 and 8 respectively in Supplementary material.

The top four provinces with the most infectious diarrhea cases are further observed (shown in Fig. 9). Compared with social factors, the meteorological factors contribute to diarrhea morbidity with greater impacts. The vapor pressure is the crucial feature and mainly affects children with ages between 2 and 9 in Guangdong province. The temperature has a primary influence on middle aged people with ages between 40-65 in Zhejiang province. Meanwhile, the junior are more vulnerable to vapor pressure while the senior are more sensitive to temperature in Anhui and Shandong provinces. Although the social factors have a weaker connection with diarrhea, some factor still has strong influence on some age groups. The quantity of health technicians has considerable effect on people with ages between 25–35 in Guangdong province. Generally speaking, the quantity of health technicians and the amounts of wastewater discharge affect people of 5–45 years old in Zhejiang province. The number of elderly people infected by diarrhea in Anhui province is connected with the quantities of health technicians, health supervision institutes and the amount of wastewater discharge. The social factors seem to contribute much less in Shandong province.

## Discussion

Our observations illustrate the vulnerability of children under 5 years old to infectious diarrhea. This group not only accounts for almost half of infectious diarrhea cases, but also is much more sensitive to pernicious exposure. In addition, our findings confirm the distinctive seasonal pattern of infectious diarrhea cases in the mainland of China while demonstrate the association between climate variation and epidemiological morbidity. In specific, we take different age groups and provinces into consideration to illustrate the change of diarrheal morbidity over time. Meteorological and social factors have been applied for prediction of number of diarrhea cases. The effects of respective factors are analyzed in different provinces, respectively. Furthermore, the influence of those factors on different age groups are evaluated, which underscore the potential use of those factors on future diversified public health management.

### Infectious diarrhea prevention for children under 5 years old

Infectious diarrhea is quite common in infancy and early childhood^65^. Children’s diarrhea is usually caused by a viral infection^66^ and is likely to damage their growth, electrolyte, and nutrient balance^67^. More seriously, episodes of diarrhea may predispose to pneumonia in undernourished children^68^, which triggers further damage to children’s health.

Numerous countermeasures have been proven to effectively reduce the high burden of infectious diarrhea in children. This includes rotavirus vaccine^69^, improvement of zinc nutriture^70^, lactobacillus therapy^71^, and dietary counseling^72^.

Our study suggests that raising the number of health technicians and improving the way of treating feces could be effective for reducing the number of children infected with diarrhea, which also agrees with some existing researches. For instance, Stephen et al.^73^ found out that participant of field workers at promoting hand washing among neighborhoods reduces the incidence of infectious diarrhea. Jai et al.^74^ showed that insecticide spraying may reduce diarrhea in children in a region where there were clear connection with fly numbers and associated diarrhea. Although the authors did not further investigate the origin of flies, it seems reasonable to recognize the relationship between the appearance of flies and the way how the feces are treated.

### Meteorological factors and their impacts on infectious diarrhea

Our observation shows that the total case of infectious diarrhea mainly increase from May to September, when the temperature and the relative humidity of the whole country increase significantly.

When considering climate-related diarrhea among different sub-population, some researches suggest that children and older adults be the most susceptible to diarrhea caused by climatic variation^75^. Both the intensity and frequency of climate changes have significant effects on childhood diarrhea^76^. Furthermore, the climate effect on infectious diarrhea for men, teenagers, and young adults (10–29 years) were higher than those for other populations^77^. Our study replenish those researches. We found that for children under 5 years old, the infectious diarrhea morbidity is apparently distinct among different seasons. And the number of young adults (20–35 years) with infectious diarrhea raises evidently in summer and early autumn, which may be caused by the lifestyle of youths but not confirmed.

Not only the divergent sub-population, but also the divergent sub-locations show a distinctive relevance with the climate-related diarrhea. For instance, a study in Wuhan (the capital of Hubei province) suggests that the central districts have high risk compared to other areas^13^. In our study, the various locations are categorized by province. Our finding reveals that coastal provinces in central and eastern China have higher numbers of infectious diarrhea cases, among which Guangdong province has the highest number of infectious diarrhea. The high morbidity of infectious diarrhea in those coastal provinces may be explained by relatively frequent contact of seafoods, but this needs further exploration.

The conjunction between infectious diarrhea and meteorological conditions may be explained by pathogens activity. Infectious diarrhea can be easily caused by microorganisms, which are closely dependent on the environmental situation^78^. Among those microorganisms, rotavirus, calicivirus, enteropathogenic and enterotoxigenic E. coli cause more than half of all diarrheal deaths in children under 5 years in the world^79–81^. The host, which those microorganisms parasitize, is directly or indirectly influenced by meteorological conditions. Microorganisms vary in the sensitivity to alterations in temperature, humidity, oxygen, light and nutrients^82–84^, which leads to different survival and reproduction rates outside of the host. Subsequently, the appropriate temperature triggers expression of the related genes inside a host^85–87^, which results in a successful infection of a host. Variations in rainfall and temperature also affect the possibility of fecal contamination^10,87^, which is an immediate cause of infectious diarrhea^48,88^. Heavy rainfalls and floods alter human exposure patterns and lead to population displacement, with a variety of resultant health impacts^87,89,90^.

Since there exists a distinct connection between meteorological conditions and infectious diarrhea, it seems natural that meteorological factors can be applied for infectious diarrhea prediction. As a matter of fact, lots of statistic models have been utilized for infectious diarrhea prediction, such as linear regression model^57,91^, multiple regression model^92^, poisson regression model^21,75,93^ and spatial panel regression model^94^. However, traditional statistic models encounter inherent flaws when approximating complicated correlation. In order to fit the inter-relation between meteorological conditions and infectious diarrhea in a better way, we exploit the Random Forest Algorithm in our study. The Random Forest Algorithm is an “ensemble learning” algorithm, which was first proposed in 2001 by Breiman. It was then widely applied in multiple domains and was considered one of the most powerful machine learning algorithms^95–98^. As a relatively new algorithm, it has been seldom employed in the field of public health. Our study shows the great potential of the Random Forest Algorithm in predicting the case number of infectious diarrhea.

### Social factors and their impacts on infectious diarrhea

The social factors that we investigated are classified into 4 categories. They are resource of public health, safety of water, fecal contamination and local forest coverage. As shown in Fig. 6, the resource of public health and safety of water play more important roles in affecting diarrhea, compared to fecal contamination and local forest coverage.

The resource of public health, which are respectively the quantity of centers for disease control and prevention (CDCP), health supervision institute (HSI) and health technicians (HT), has obvious influence on the whole age groups. The positive influence of those factors may owe to their improvement of sanitation and hygiene, as infectious diarrhea could be easily caused by contaminated food^43,99–101^, bad sanitation environment^54^ and poor health situation^45,46^. Those risk factors of infectious diarrhea can be mitigated by health intervene, like a good habit of hand washing^54,102^, appropriate food interventions^47,102^, a relatively rapid diagnose/solution^103^ and more medical technicians^41^. Increasing the quantity of CDCP, HSI and HT provide more health education for public and more professional treatments of patients^103,104^. Thus more resource of public health results in more effective diarrhea prevention.

As shown in Fig. 6, compared to other kinds of social factors, the safety of water has considerable impact on incidence of diarrhea, especially to the elderly. The major concern about the water safety is water pollution^61^. Existing researches demonstrate that not only the polluted water for drinking, but also the polluted water for using can bring infectious diarrhea^42,52,53^. The water pollution may trigger diarrhea through pathogens contamination and cause physical function degeneration. A review study illustrates that the most common polluters of water are pathogens (bacteria, viruses and protozoas)^61^. Those pathogens could lead to various kinds of infectious diarrhea. On the other hand, water pollution injury is likely to cause diseases like rheumatic, vascular diseases, myocardial infarct and nervous system damage, especially to the elderly^105^. Those diseases could make the elderly more vulnerable to diarrhea^106^.

The fecal contamination is represented by the weight of feces that are harmfully treated. The fecal contamination may result in diarrhea through pathogens contamination directly or indirectly. For instance, there are studies showing that the fecal contamination is associated with viral and bacterial infection^51,54^. Some researches demonstrate that the fecal contamination lead to water pollution^22,47^, which may further bring about diarrhea infection.

In contrast to other social factors, forest coverage has less impact on infectious diarrhea. The relationship between forest coverage and diarrhea may result from the the influence on environment from forest. For instance, there is research illustrating that forest contributes to diarrhea prevention by improving water quality^55^. Another study shows that heatwaves might promote the occurrence of cholera, but this connection could be modified by forest coverage^24^.

Our study shows that different social factors have different effects on specific age groups. Those findings shed new lights to infectious diarrhea prevention and may promote more flexible and efficient disease control measurement. For instance, our research indicates that the number of health technicians plays a more important role to children under 5 years old, youth with age between 15 and 30, and the elderly over 60 years old. A connection has been observed between the weight of feces that are harmfully treated and monthly numbers of infectious diarrhea cases of the old as well as children under 5 years old. Based on those findings, health technicians should put much more attention to those two age groups when considering infectious diarrhea prevention, which may bring about more accurate disease control as well as rational and effective management of human resource. Meanwhile, the weight of feces that are harmfully treated should be brought to the forefront.

Figure 9 further demonstrates that the impact of social factors differ in different provinces. For instance, the social factors have a strong connection with diarrhea in Guangdong province than in Shandong province. The regional difference may result from socio-demographic variousness^107^. Although the mechanism remains unclear, it seems significant that geographical locations should be put on more attention when considering infectious diarrhea^108^.

Figure 9 also shows that social factors contribute to diarrhea morbidity, but not in a dominant way. This conclusion coincides with some existing researches^13,37^. Furthermore, some study reveals that the impact social factors have on diarrhea remains unstable^107^. Further investigations are needed to explore major mechanisms underlying the association between infectious diarrhea and certain social factors.

## Methods

### Diarrhea data

In China, infectious diarrhea (excluding cholera, dysentery, typhoid, and paratyphoid) is an intestinal infectious disease with diarrhea and/or vomiting as the main symptom. Monthly aata on infectious diarrhea during 2005–2017 in mainland of China were collected from the Nationwide Notifiable Infectious Diseases Reporting Information System (NIDRIS)^109^. The information included time, age, and date of onset. The data are publicly available.

### Meteorological data

Monthly climate data were obtained from the National Meteorological Information Center of China (http://data.cma.cn/), including precipitation, wind speed, temperature, vapor pressure, and relative humidity. The data are publicly available.

### Social factors data

Monthly social factors data were obtained from the Chinese National Bureau of Statistics (http://www.stats.gov.cn), containing the quantity of centers for disease control and prevention, the quantity of health supervision institute, the quantity of health technicians, the amounts of wastewater discharge, chemical oxygen demand emissions, nitrogen output, the weight of Feces that are harmfully treated and the forest coverage rate. The data are publicly available.

### Data analysis

In our study, the Random Forest Algorithm was applied for data analysis, whose standard procedure is repeated as follows^110^:

1. Draw n$_{tree}$ bootstrap samples from the original data.
2. For each of the bootstrap samples, grow an unpruned classification or regression tree, with the following modification: at each node, rather than choosing the best split among all predictors, randomly sample m$_{try}$ of the predictors and choose the best split from among those variables. (Bagging can be thought of as the special case of random forests obtained when m$_{try}$ = p, the number of predictors.)
3. Predict new data by aggregating the predictions of the n$_{tree}$ trees (i.e., majority votes for classification, average for regression).

An estimate of the error rate can be obtained, based on the training data, by the following:

1. At each bootstrap iteration, predict the data not in the bootstrap sample (what Breiman calls “out-of-bag”, or OOB, data) using the tree grown with the bootstrap sample.
2. Aggregate the OOB predictions. (On the average, each data point would be out-of-bag around 36% of the times, so aggregate these predictions.) Calculate the error rate, and call it the OOB estimate of error rate.

## Supplementary Information

Supplementary Table 1.

Supplementary Table 2.

Supplementary Table 3.

Supplementary Table 4.

Supplementary Table 5.

Supplementary Table 6.

Supplementary Table 7.

Supplementary Table 8.

Supplementary Table 9.

Supplementary Table 10.

Supplementary Table 11.

Supplementary Table 12.
